# Supplementary material for: Toward developing a sustainability index for the Islamic Social Finance program: An empirical investigation
Source: PLoS One. 2022 Nov 22;17(11):e0276876. doi: 10.1371/journal.pone.0276876 (PMC9681090; doi:10.1371/journal.pone.0276876)
Supplement: S1 File — (DOCX) [file pone.0276876.s002.docx]

**Minimal Data (Questionnaire)**

**QUESTIONNAIRE**

***Towards Developing Socio-Economic Sustainability Index: An Empirical Study of Islamic Social Finance Program***

Respondent Name :

Institution :

Position :

No. Hp (WA) :

Email address :

Filling date :

Respondent's signature :

**Introduction**

Islamic Social Finance significantly contributes to sustainable development, especially in solving problems of poverty and inequality. One of the Islamic Social Finance programs is an empowerment program that aims to increase the beneficiaries' independence and quality of life. Therefore, Islamic Social Finance has excellent potential. Based on data from the Indonesian Waqf Agency, the potential for national cash waqf reaches IDR 180 trillion per year, while the zakat collection has the potential to reach IDR 327.6 trillion (BAZNAS, 2021). However, the realization of zakat and waqf collection only got 3% of the total potential of zakat and 0.21% of the full potential of cash waqf. This fact indicates a severe problem in the governance of Islamic social finance. The quality of Islamic Social Finance control can be improved by establishing performance measurement standards, especially for measuring the success and sustainability of Islamic social fund management programs. This measurement is crucial to determine whether the Islamic social fund (utilization program) provided to the beneficiaries can create socio-economic sustainability so that the beneficiaries can achieve prosperity. In Islamic Social Finance, sustainability relates to the ability of beneficiaries to transform. This research aims to build an index of the success and sustainability of Islamic Social Finance programs (utilization programs) by emphasizing the aspects of success and sustainability. The questionnaire below aims to build an index of the success and sustainability of Islamic Social Finance programs (utilization programs) by analyzing the dimensions, aspects, and indicators that affect the program's sustainability.

**Output**

This research aims to build an index of success and sustainability of the Islamic Social Finance program (utilization program) by analyzing the priority values of each dimension, aspect, and indicator formulated.

**Instructions**

This questionnaire is the primary data collection for the research "Towards Developing Sustainability Index: An Empirical Study of Islamic Islamic Social Finance Program". This questionnaire is designed to assess expert respondents, including representatives from practitioners, academics, regulators, associations, supervisors, and community leaders. There are no right or wrong answers, but this study intends to compare the priority weights of each formulated dimension, aspect, and indicator. The rating scale will be explained as follows:

1 = Not important/Relevant/Influential

2-3 = Less important/Relevant/Influential

4-5 = Important/Relevant/Influential

6-7 = Very Important/Relevant/Influential

8-9 = Very very important/Relevant/Influential

**UTILIZATION PROGRAM SUSTAINABILITY FACTORS**

Q1. Sort the UTILIZATION PROGRAM SUSTAINABILITY FACTORS below (ranked 1 to 2)?

Q2. How important are the following UTILIZATION PROGRAM SUSTAINABILITY FACTORS in supporting the success and sustainability of the utilization program (scale 1 to 9)?

| **No.** | **Factor** | **Rank (Q1)** | **How Important (Q2)** | **Definition** |
| --- | --- | --- | --- | --- |
| 1. | Internal |  |  | Internal factors are all factors directly involved in program management. In this study, internal factors include stakeholders who directly ensure the program's sustainability. These stakeholders include Islamic Social Finance institutions, beneficiaries, and supervisors. |
| 2. | External |  |  | External factors are all factors that are not directly involved in program management. In this study, external factors include associations, academics, regulators, and the government. |

**Rating Scale How Important (Q2)**

| **1** | **2** | **3** | **4** | **5** | **6** | **7** | **8** | **9** |
| --- | --- | --- | --- | --- | --- | --- | --- | --- |
| **Not important/Relevant/ Influential** |  | **Less important/Relevant/Influential** |  | **Important/Relevant/ Influential** |  | **Very important/Relevant/Influential** |  | **Very very important/**  **Relevant/Influential** |

**STAKEHOLDER DIMENSION ANALYSIS IN SUPPORTING THE ACHIEVEMENT OF SUSTAINABILITY USAGE PROGRAM**

Q1. In order which STAKEHOLDERS are most instrumental in achieving the success and sustainability of the utilization program (ranked 1 to 8)?

Q2. Which STAKEHOLDER has the most role in supporting the success and sustainability of the utilization program (scale 1 to 9)?

| **CODE** | **Stakeholder** | **Rank (Q1)** | **How important (Q2)** |
| --- | --- | --- | --- |
|  | Islamic social finance institutions |  |  |
|  | Beneficiary |  |  |
|  | Benefit recipients |  |  |
|  | Supervisor |  |  |
|  | Association |  |  |
|  | Academics |  |  |
|  | Regulator |  |  |
|  | Government |  |  |

**Rating Scale How Important (Q2)**

| **1** | **2** | **3** | **4** | **5** | **6** | **7** | **8** | **9** |
| --- | --- | --- | --- | --- | --- | --- | --- | --- |
| **Not important/Relevant/ Influential** |  | **Less important/Relevant/Influential** |  | **Important/Relevant/ Influential** |  | **Very important/Relevant/Influential** |  | **Very very important/**  **Relevant/Influential** |

**ASPECT ANALYSIS IN SUPPORTING THE ACHIEVEMENT OF SUSTAINABLE UTILIZATION PROGRAM**

Q1. In order which ASPECTS are most instrumental in achieving the success and sustainability of the utilization program (ranked 1 to 19)?

Q2. Which ASPEK plays a very important role in supporting the success and sustainability of the utilization program (scale 1 to 9)?

| ***Stakeholder*** | **CODE** | **Aspect** | **Rank (Q1)** | **How important (Q2)** |
| --- | --- | --- | --- | --- |
| **Islamic Social Finance Institution** | A.1 | Human Resources |  |  |
|  | A.2 | Planning |  |  |
|  | A.3 | Collection |  |  |
|  | A.4 | Management |  |  |
|  | A.5 | Utilization |  |  |
|  | A.6 | Reporting |  |  |
|  | A.7 | Monitoring and Evaluation |  |  |
| **Beneficiary** | B.1 | Involvement |  |  |
| **Benefit recipients** | C.1 | Commitment and Engagement |  |  |
|  | C.2 | Spirituality |  |  |
|  | C.3 | Quality of Life |  |  |
| **Supervisor** | D.1 | Supervision |  |  |
|  | D.2 | Evaluation |  |  |
| **Association** | E.1 | Coordination |  |  |
|  | E.2 | Cooperation |  |  |
| **Academics** | F.1 | Socialization |  |  |
| **Regulator** | G.1 | Regulatory Support |  |  |
| **Government** | H.1 | Infrastructure Support |  |  |
|  | H.2 | Other Support |  |  |

**Rating Scale How Important (Q2)**

| **1** | **2** | **3** | **4** | **5** | **6** | **7** | **8** | **9** |
| --- | --- | --- | --- | --- | --- | --- | --- | --- |
| **Not important/Relevant/ Influential** |  | **Less important/Relevant/Influential** |  | **Important/Relevant/ Influential** |  | **Very important/Relevant/Influential** |  | **Very very important/**  **Relevant/Influential** |
