# Supplementary material for: Toward developing a sustainability index for the Islamic Social Finance program: An empirical investigation
Source: PLoS One. 2022 Nov 22;17(11):e0276876. doi: 10.1371/journal.pone.0276876 (PMC9681090; doi:10.1371/journal.pone.0276876)
Supplement: Attachment — Submitted filename: Response to Reviewer.docx [file pone.0276876.s003.docx]

| **Manuscript ID** | PONE-D-22-20909 |
| --- | --- |
| **Title** | Toward Developing a Sustainability Index for the Islamic Social Finance Program: An Empirical Investigation |

| **No** | **Reviewer’s Comment** | **Respond** | **Page** | **Reviewer’s/Editor’s Comment** |
| --- | --- | --- | --- | --- |
| 1 | Please ensure that your manuscript meets PLOS ONE's style requirements, including those for file naming. The PLOS ONE style templates can be found at  [https://journals.plos.org/plosone/s/file?id=wjVg/](https://journals.plos.org/plosone/s/file?id=wjVg/PLOSOne_formatting_sample_main_body.pdf)  PLOSOne_formatting_sample_main_body.pdf and  [https://journals.plos.org/plosone/s/file?id=ba62/ f](https://apc01.safelinks.protection.outlook.com/?url=https%3A%2F%2Fjournals.plos.org%2Fplosone%2Fs%2Ffile%3Fid%3Dba62%2FPLOSOne_formatting_sample_title_authors_affiliations.pdf&data=05%7C01%7Canidah%40utem.edu.my%7Cf40ccf9920e541e1136208da89643eb3%7C677eeb56f2df46759e0cccd600a91580%7C0%7C0%7C637973360905383022%7CUnknown%7CTWFpbGZsb3d8eyJWIjoiMC4wLjAwMDAiLCJQIjoiV2luMzIiLCJBTiI6Ik1haWwiLCJXVCI6Mn0%3D%7C3000%7C%7C%7C&sdata=TbeWXDkHUpUTLvyBUxJFDGhkhEXuiQ3GOM%2BgSNuWV%2Fk%3D&reserved=0)  PLOSOne_formatting_sample_title_authors_affiliations.pd | Thank you for your comment  We've made improvements to our manuscript with the PLOS ONE template | All Page |  |
| 2 | Please include your full ethics statement in the ‘Methods’ section of your manuscript file. In your statement, please include the full name of the IRB or ethics committee who approved or waived your study, as well as whether or not you obtained informed written or verbal consent. If consent was waived for your study, please include this information in your statement as well | Thank you for your comment  We have included the ethics committee in the method section in the “Data and Samples” subsection highlighted in yellow. In addition, we also include an ethics committee in acknowledgment  We explain that this research is supervised and monitored by the Center of Research and Publication (3P) as ethics committee at Faculty level and Institute for Research and Community Service (LPPM) Universitas Airlangga at University level | Section “Material and Method”, sub section “data and samples” page 11 on the last paragraph highlighted in yellow |  |
| 3 | PLOS ONE does not copy edit accepted manuscripts ([https://journals.plos.org/plosone/s/criteria-for-publication#loc-5](https://journals.plos.org/plosone/s/criteria-for-publication#loc-5)). To that effect, please ensure that your submission is free of typos and grammatical errors. | Thank you for your comment  We have proofread our manuscript to ensure there are no typographical and grammatical errors |  |  |
| 4 | In your Data Availability statement, you have not specified where the minimal data set underlying the results described in your manuscript can be found. PLOS defines a study's minimal data set as the underlying data used to 5reach the conclusions drawn in the manuscript and any dditional data required to replicate the reported study findings in their entirety. All PLOS journals require that the minimal data set be made fully available. For more information about our data policy, please see <http://journals.plos.org/plosone/s/data-availability>.  Upon re-submitting your revised manuscript, please upload your study’s minimal underlying data set as either Supporting Information files or to a stable, public repository and include the relevant URLs, DOIs, or accession numbers within your revised cover letter. For a list of acceptable repositories, please see <http://journals.plos.org/plosone/s/data-availability#loc-recommended-repositories>. Any potentially identifying patient information must be fully anonymized.  Important: If there are ethical or legal restrictions to sharing your data publicly, please explain these restrictions in detail. Please see our guidelines for more information on what we consider unacceptable restrictions to publicly sharing data: <http://journals.plos.org/plosone/s/data-availability#loc-unacceptable-data-access-restrictions>. Note that it is not acceptable for the authors to be the sole named individuals responsible for ensuring data access.  We will update your Data Availability statement to reflect the information you provide in your cover letter. | Thank you for your comment  The data we used are primary data from FGDs, in-depth interviews, and Questionnaires distributed among experts in Islamic institutions such as Zakat officers.  Therefore, at least the data can be accessed by contacting the corresponding author”. As information for readers, we have provided related information where data can be accessed in the methods section and appendix. | Section “Material and Method”, sub section “data and samples” page 9 on the first paragraph |  |
| 5 | Please ensure that you refer to Figure 2 in your text as, if accepted, production will need this reference to link the reader to the figure | Thank you for your comment  We have referenced Figure 2 in the text | Page 7 on the sub section “method and technique analysis” on the 2rd paragraph |  |
| 6 | Please review your reference list to ensure that it is complete and correct. If you have cited papers that have been retracted, please include the rationale for doing so in the manuscript text, or remove these references and replace them with relevant current references. Any changes to the reference list should be mentioned in the rebuttal letter that accompanies your revised manuscript. If you need to cite a retracted article, indicate the article’s retracted status in the References list and also include a citation and full reference for the retraction notice. | Thank you for your comment  We have identified our citations and separated which journals are still continuing with scope and which are journals that have been discontinued.  We decided to delete the discontinued journals, and some of them we replaced with other citations  However, I would like to inform you that there is one SCOPUS discontinued paper but we are still referring to it due to its high relevancy. The paper was written and published by one of our authors, and also considers the relevance of the paper to our paper. The papers are: Robani A, Salih K. Positioning Islamic gift economy for sustainable development at the local level. Humanities and Social Science Reviews. 2018;6(2):111–20  In addition, there are several national journals and international journals (with other indexations) that we continue to include considering their relevance | All Manuscript |  |
| 7 | Is the manuscript technically sound, and do the data support the conclusions?  The manuscript must describe a technically sound piece of scientific research with data that supports the conclusions. Experiments must have been conducted rigorously, with appropriate controls, replication, and sample sizes. The conclusions must be drawn appropriately based on the data presented.  Reviewer #1: Yes  Reviewer #2: Partly | Thank you for your review |  |  |
| 8 | Has the statistical analysis been performed appropriately and rigorously?  Reviewer #1: N/A  Reviewer #2: Yes | Thank you for your review |  |  |
| 9 | Have the authors made all data underlying the findings in their manuscript fully available?  The [PLOS Data policy](https://apc01.safelinks.protection.outlook.com/?url=http%3A%2F%2Fwww.plosone.org%2Fstatic%2Fpolicies.action%23sharing&data=05%7C01%7Canidah%40utem.edu.my%7Cf40ccf9920e541e1136208da89643eb3%7C677eeb56f2df46759e0cccd600a91580%7C0%7C0%7C637973360905383022%7CUnknown%7CTWFpbGZsb3d8eyJWIjoiMC4wLjAwMDAiLCJQIjoiV2luMzIiLCJBTiI6Ik1haWwiLCJXVCI6Mn0%3D%7C3000%7C%7C%7C&sdata=gFbD9fEEmfFoC77G%2B4oSvOga62jdyQpwPPi9pYJSHKw%3D&reserved=0) requires authors to make all data underlying the findings described in their manuscript fully available without restriction, with rare exception (please refer to the Data Availability Statement in the manuscript PDF file). The data should be provided as part of the manuscript or its supporting information, or deposited to a public repository. For example, in addition to summary statistics, the data points behind means, medians and variance measures should be available. If there are restrictions on publicly sharing data—e.g. participant privacy or use of data from a third party—those must be specified.  Reviewer #1: Yes  Reviewer #2: Yes | Thank you for your review |  |  |
| 10 | Is the manuscript presented in an intelligible fashion and written in standard English?  PLOS ONE does not copyedit accepted manuscripts, so the language in submitted articles must be clear, correct, and unambiguous. Any typographical or grammatical errors should be corrected at revision, so please note any specific errors here.  Reviewer #1: Yes  Reviewer #2: No | Thank you for your review  We have proofread our manuscript | All page |  |
| 11 | Reviewer #1: This articles is very interesting and beautifully written, however few grammatical, punctuations and syntax issues were seen. This paper can be accepted. I recommend to add the time series of different policies and their impact on social financing program. In addition, please elaborate more on exiting system loops and holes and their long-term consequences on financing. | Thank you for your review  1. We have proofread our manuscript  2. We have added short, medium, and long term practical implications as recommendations for what policies should be taken by ISF Institutions to have a positive impact on aid recipients. | Page 19 section “practical implications” |  |
| 12 | Reviewer #2: 1. Please rephrase the objectives of the study. I believe it will help readers to clearly understand the objective of the study. 2. A summary paragraph of literature review will help readers to understand the overall depiction of literature. 3. Please add more details in the discussion section. 4. In the conclusion section, please add limitations of the study and future recommendations. Please also provide clear policy implications. I suggest the author to write implications one by one so readers can easily read it. Overall, the author should review the paper, remove all grammatical and punctuations related errors. | Thank you for your review  1. We have paraphrased the research objectives in the abstract and introduction sections  2. We have added a summary of literature review (in the last sentence or the last paragraph for every sub section)  3. We have added details in the discussion section on the last paragraph  4. We have written the limitations and recommendations for further research in the conclusion. Regarding implication, we separate the conclusion from the implication section. However, we have refined the implications of this study by explaining what roles each stakeholder should take. | 1. Page 1 and 2, abstract and introduction highlighted in yellow  2. Page 3 – 7 Section “Literature review”  3. Page 18 “Discussion” highlighted in yellow  4. Page 20 “Conclusion” on the last paragraph highlighted in yellow |  |

**CERTIFICATE OF PROOFREADING**
